# Connectomics and the neural basis of behaviour

**DOI:** 10.1016/j.cois.2022.100968

**Published:** 2022-09-13

**Authors:** Dana S Galili, Gregory SXE Jefferis, Marta Costa

**Affiliations:** 1 Neurobiology Division, MRC Laboratory of Molecular Biology, Cambridge CB2 0QH, UK; 2 Drosophila Connectomics Group, Department of Zoology, University of Cambridge, Downing Street, Cambridge CB2 3EJ, UK

## Abstract

Methods to acquire and process synaptic-resolution electron-microscopy datasets have progressed very rapidly, allowing production and annotation of larger, more complete connectomes. More accurate neuronal matching techniques are enriching cell type data with gene expression, neuron activity, behaviour and developmental information, providing ways to test hypotheses of circuit function. In a variety of behaviours such as learned and innate olfaction, navigation and sexual behaviour, connectomics has already revealed interconnected modules with a hierarchical structure, recurrence and integration of sensory streams. Comparing individual connectomes to determine which circuit features are robust and which are variable is one key research area; new work in comparative connectomics across development, experience, sex and species will establish strong links between neuronal connectivity and brain function.

## Rapid progress in connectomics

Connectomics has developed extremely rapidly in the last five years and some of the most impactful developments have been in insect brains [1]. Increased electron-microscopy (EM) imaging speed and improvements in automated segmentation mean that complete synaptic-resolution wiring diagrams of the size of a whole fruit fly brain are now feasible. Although many insect species are now potential targets for EM connectomics, progress so far has mostly been in *Drosophila melanogaster*, which is therefore the focus of this review.

Improvements to sample preparation, and serial-section transmission [••2] and focused ion beam scanning EM imaging [••3,^4^] have made pipelines more robust. This, together with optimised image alignment, has improved automated segmentation methods for neurons [••3,^5^,^6^] and synapses [••3,^7^,^8^]. A newly generated EM volume can now be densely populated with connectivity and neuronal morphologies, many of which may be immediately identifiable.

Despite this progress in automation, manual proofreading to remove false merges between neurons and find missed branches remains essential. The proofreading effort for the central brain of *Drosophila* (∼40 000 neurons) has been reduced from order 2000 to ∼30 person-years. But this is still a major effort, depending on organised proofreading projects, which range from completely in-house to community-distributed. Compared with the first similar effort [9], the speed-up offered by automated segmentation allows research groups to contribute weeks to months of effort while deriving significant insights. Nevertheless, so far, only dedicated and highly coordinated proofreading teams have delivered complete connectomes of tens of thousands of neurons, typical of the adult fly brain and ventral nerve cord (VNC) [••3].

**Figure 1:**
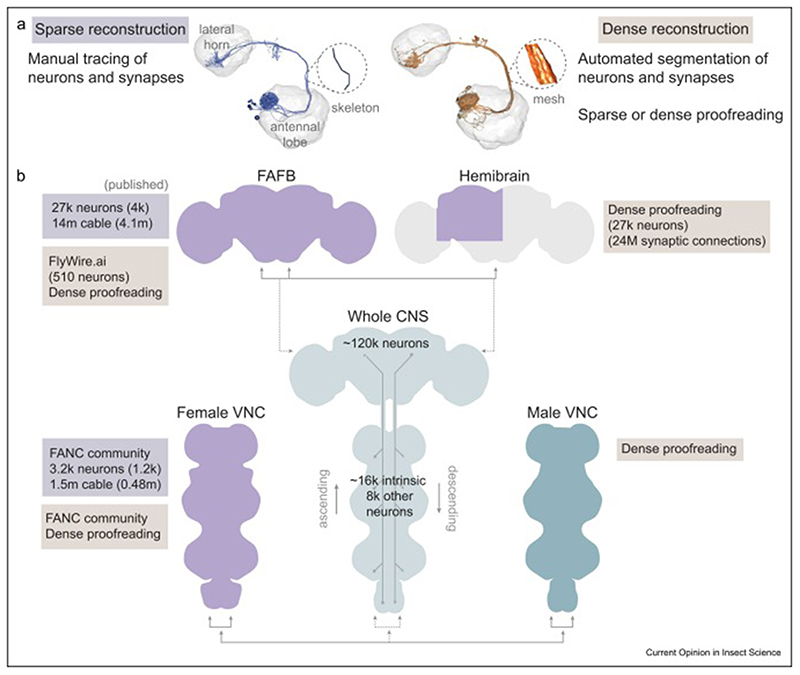
*Adult Drosophila melanogaster* EM connectomics volumes. **(a)** Comparison between sparse and dense EM reconstruction and proofreading. The reconstructed olfactory-projection neurons DA1 lPNs are shown as an example for sparse (purple, data from https://fafb.catmaid.virtualflybrain.org/) [79] and dense reconstruction (orange, data from https://neuprint.janelia.org/) [••3,•17]. The neuron format (skeleton or volumetric mesh) is independent of the reconstruction being sparse or dense. The examples shown are constrained by the publicly available data. **(b)** Adult *Drosophila melanogaster* EM datasets available and future ones (whole central nervous system (CNS)). Comparisons possible within (across hemispheres) and between them (across individuals) are indicated by arrows. Dashed lines indicate future comparisons. For each project, the number of reconstructed neurons, total reconstructed cable length or synaptic connections is shown. Numbers in brackets are for published data. For the full adult fly brain (FAFB) and the female VNC sparse reconstructions, the number of unpublished neurons captures any skeleton with more than 100 nodes. The total reconstructed cable includes any skeleton with more than two nodes.

The pioneer connectomics volume in Drosophila was the larval first instar (L1) central nervous system (CNS) [9]. It sparked analysis in several areas from learning and memory [10,•11] to developmental mechanisms [12,•13], often linking the observed connectivity with behaviour (reviewed in Ref. [14]). Seven subvolumes comprising an adult olfactory glomerulus allowed some initial subcellular analysis and morphological diversity discovery [15]. The first adult whole-brain volume evolved from sparse manual tracing [16] to dense automated reconstruction followed by sparse proofreading [••5] (Figure 1). The release of the dense connectome for a partial adult female brain [••3] was the first resource allowing researchers instant access to a finished insect connectome. Together, both volumes allow comparisons between individuals and across three hemispheres [17]. Since then, a sparse female VNC reconstruction has been published [••2] and a dense autosegmentation will be released soon (personal communication, W. Allen Lee and J. Phelps). A densely reconstructed male VNC is also expected shortly (personal communication, S. Berg, G. Rubin, G. Card and G. Jefferis).

Reconstructing whole volumes provides immediate benefits beyond the analytical power. Proofreading and neuron identification are significantly accelerated by cross-comparisons. Although generating a connectome is still resource-intensive, current progress [•18] means that central nervous system (CNS) volumes for both sexes should become available within 2–3 years. Mapping dimorphic neurons and connections between male and female central nervous system (CNS) volumes could explain observed differences in behaviour (Box 1).

**Figure 2:**
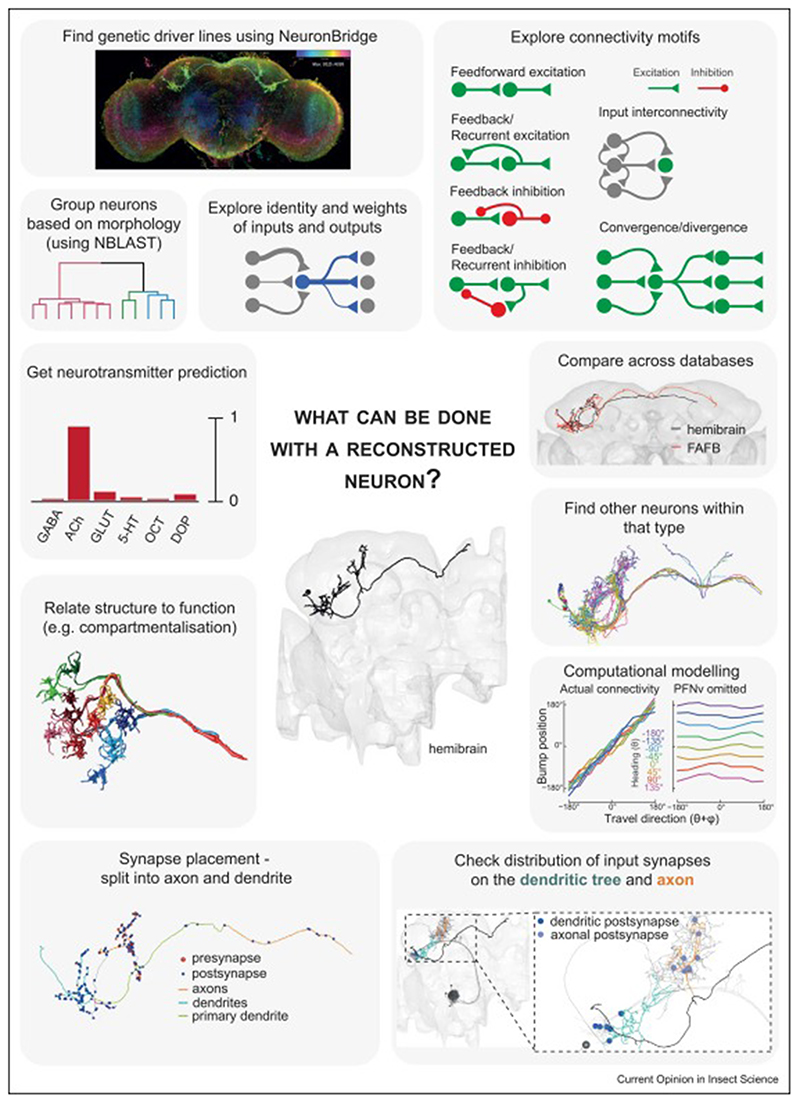
What can be done with a reconstructed neuron? Centre: Visualisation of the hemibrain surface and a single aSP-g2 neuron (hemibrain bodyid=641278400). From top left, going clockwise: · Find genetic driver lines using NeuronBridge [••27,•30]. Use colour-depth search or PatchPerPixMatch 29 to find driver lines expressing the neuron of interest. · Group neurons based on morphology (using NBLAST 36). This is used to define types. · Explore identity and weights of inputs and outputs. When exploring connectivity, it is useful to collapse by type and check not only the number of synapses but also the proportion of synapses from or to connecting neurons. · Explore connectivity motifs (examples shown). · Compare across databases — how stereotypic is the neuron? Are parts missing between datasets? Here, the hemibrain aSP-g2 neuron (black) and the homologous full adult fly brain (FAFB) aSP-g2 neuron (red) are both plotted in full adult fly brain (FAFB) coordinates. Notice the hemibrain neuron is truncated. Morphological comparisons can also be made against neurons from light microscopy (LM) datasets, for example, FlyCircuit (www.flycircuit.tw) [80]. · Find other neurons within that type, either manually identifying neurons travelling together within a tract, or by comparing similarity within a set of known neurons (using NBLAST 36). Many neurons on the hemibrain dataset were curated by type, so are easily found. Visualisation of all aSP-g neurons within the hemibrain dataset. · Computational modelling of network function using connectivity data. In this example, a model of fan-shaped body (FB) circuit using actual connectivity information showed a strong correlation between bump position and travel direction (left panel). This correlation is lost when specific connections are eliminated from the model (right panel, PNFv neurons omitted) (image kindly provided by R. I. Wilson, author of 20. · Check distribution of input synapses on the dendritic tree (green) and axon (orange). It is useful to plot neurons of interest and their partners to examine whether input connectivity is axo-dendritic feedforward, axo-axonic or other. Strong inputs to a hemibrain aSP-g neuron (black) in each subcompartment are plotted: olfactory projection neuron DA1 lPN synapses on dendrites (blue), and local neuron synapses on axons (purple). Dotted line shows a zoomed-in section in inset. Additionally, the spatial distribution of synapses along the dendritic tree might affect the functional connectivity strength, as was suggested for MBONs [39] and EPG neurons [61]. · Synapse placement — split a neuron into axon and dendrite. Flow-centrality analysis with natverse [79] produced an axon and dendrite split, showing pre- (red) and postsynaptic (blue) connections. · Relate structure to function, for example, compartmentalisation. In this Amacrine cell, thin, loopy branches provide electrotonic separation between compartments. This morphology suggests the neuron performs local computations, which was confirmed functionally [81] (image used with permission from publisher). · Get neurotransmitter prediction [•19].

Wiring diagrams can generate and test behavioural hypotheses or help interpret experimental results. This approach is becoming more widespread and we showcase some examples below and in Figure 2. These insights are enriched by augmenting the connectome with other types of data. Machine learning approaches can predict neurotransmitter identity from EM data with high accuracy [•19]. Further work could broaden the applicable neurotransmitters and compare across datasets. Nevertheless, this signal context already aids in interpreting connectivity features and supporting functional observations [•20]. Single-cell transcriptomic approaches are increasingly prevalent and more comprehensive with whole adult data now available [•21–24]. The next steps could fully map these data in the central nervous system (CNS) at the resolution of connectomics cell types. Integrating developmental information onto the static connectome can also uncover valuable insights. In the larva, developmental patterns were shown to affect synaptic specificity. The interaction of spatial, hemilineage, and temporal identity rules drives the observed connectivity patterns, with related neurons more likely to share connections and partners [•13,^25^]. These refinement rules are apparent in the proprioceptive motor circuit, where first-, second- and third-order types downstream of the sensory input can each be assigned to a specific temporal-hemilineage identity that impinges on neuronal identity and connectivity [•13]. Furthermore, circuit structure is also impacted, with neuronal outputs born earlier than inputs [25].

Intersecting gene expression, developmental rules, neurotransmitter and connectivity information at the cell type level will undoubtedly strengthen the interpretation of neuronal function [26].

## Integrative connectomics

Establishing direct links between the structural connectivity information of connectomics with circuit physiology and behaviour remains a challenge. The first step requires matching morphologies between EM and lower-resolution light-level microscopy (LM) data. Methods to semi-automate this process have been developed, taking advantage of extensive light microscopy (LM) driver lines and MultiColour FlpOut libraries [••27–•30]. The success of this process strongly correlates with the amount of data available per morphology, as the range of natural variability in both EM and light microscopy (LM data), and potential developmental abnormalities need to be considered, not only to map individual neurons but to define cell types [•17,^31^] — the reproducible and recognisable unit of neuronal morphology and connectivity. Collapsing information by cell type is important as it can overcome inherent variability in biological datasets. In the hemibrain, over 5200 morphological cell types were identified, with some further broken down by connectivity. Only 20% of those were previously described [••3]. We expect that a subset of these morphological and connectivity types will be further refined or consolidated as more cross-comparisons are possible.

Additional uncertainties include the relationship between connectomics and physiological connection strengths and how neuronal firing might be impacted by variations in synapse location, these issues can only be conclusively addressed by detailed physiological measurements from many neuron types [•32]. Understanding the relationship between connectivity and activity will be of major importance in the next few years, eventually allowing us to interpret connection strengths in the context of whole circuits and behaviour, and provide a deeper understanding of natural variability. Other factors such as the influence of gap junctions, neuromodulators and glia are recognised unknowns, however harder to address with current datasets. Staining protocols, as well as automated segmentation methods are usually optimised to recognise chemical synapses and neurons, thus those other features are either not discernible in the image data or not segmented correctly, precluding a comprehensive analysis without very significant effort [33]. Focused approaches to identify these factors would necessitate changes to staining methods, resegmentation of existing datasets after algorithm optimisation or mapping markers identified by light microscopy (LM) to EM neurons [34]. Certainly, the availability of additional EM volumes will allow a more detailed analysis of stereotypy per cell type, for morphology and connectivity and further comparisons to other data types (Figure 2).

Importantly, releasing datasets and annotated connectomes freely and quickly to the community enables a shared framework to link to literature and support communication across labs. This aids the understanding of the multiple functions and contexts in which a given circuit might be acting.

## From connectomics to behaviour

### Initial studies relating connectomics to behaviour

The earliest mapping between connectome and function in *Drosophila* was in the larva, dissecting the circuit underpinning rolling escape behaviour [9]. Second-order neurons were identified in EM, that integrate combinations of nociceptive and mechanosensory signals triggering rolling, depending on their input. Further analysis of deeper circuit levels revealed that convergence of multimodal information occurred at multiple layers, in both the brain and VNC.

In the adult, EM connectivity was crucial to interpret and corroborate light microscopy (LM) and behaviour data in the olfactory system, demonstrating the interaction between innate and learned circuits for aversive memory retrieval [•35]. This work discovered key neurons in the lateral horn, a brain region implicated in sensory processing for innate behaviour, downstream of a mushroom body output neuron (MBON). Comprehensive EM reconstruction followed by quantification of morphological similarity to light microscopy (LM) data [36], allowed the unequivocal matching between connectome and genetically defined cell types [•35]. The availability of good bridging registrations between light microscopy (LM) and EM brain templates was crucial for this *in silico* mapping [37].

### Memory circuits of the mushroom body

Olfactory memories are formed through a plastic, compartmentalised and recurrent network; coincidence between odour and reinforcing signals carried by valencespecific dopaminergic neurons (DANs) changes the connection strength between mushroom body (MB) Kenyon Cells and their downstream partners, MBONs. After training with punishment or reward, flies will avoid or approach the associated odour in subsequent exposures.

EM reconstruction of larval MB circuitry unveiled dense recurrent connections between Kenyon Cells, and reciprocal connectivity between DANs and both Kenyon Cells and MBONs [10]. Extending this map revealed that punishment is indirectly conveyed to DANs from nociceptive and mechanosensory neurons, while previous experience is fed back onto many DANs by neurons downstream of MBONs [•11]. This feedback enables adaptive regulation of DANs based on experience and could serve as the basis for encoding prediction errors and expectation. Further work identified substantial convergence between MBONs and lateral horn neurons, providing an initial architecture and mechanism for integrating learned and innate behaviour [38], similar to the adult [•35].

Repeated exposure to a learned odour without expected reinforcement leads to memory extinction. Recent work in adults found that extinction is an active mechanism, in which a newly formed memory trace competes with the old one [39]. The new memory is formed by recurrent feedback from MBONs to opposite-valence DANs, whose activity serves as a new ‘reinforcement’ signal. The overall effect of the competing pathways — avoidance- and approach-promoting — is integrated and neutralised, leading to behavioural extinction. Analysing synapse location showed that inhibitory synapses from an approach-promoting MBON targeted the dendritic root of an avoidance-promoting MBON, and could therefore shunt activity from entire dendrites [39]. Following aversive learning, release of such strong inhibition would bias the MBON network towards odour avoidance. Indeed, inhibition between MBONs serves to integrate opposing memory traces after extinction. Additionally, recurrent inhibition and disinhibition between MBONs and DANs enables specific gain control with subsequent odour exposure, thereby adapting learning rules by expectation [39,••40].

A new study describes a feedforward circuit from a reward-representing MBON to DANs in other MB compartments, required for second-order learning [41]. In second-order learning, animals first learn to associate a stimulus with reinforcement and then to associate a new stimulus with the first one. The authors used hemibrain connectivity and neurotransmitter predictions to identify cholinergic interneurons downstream of reward-representing MBONs. These interneurons are activated in the initial learning phase, and excite DANs in other MB compartments. This plasticity enables a novel stimulus to acquire a similar valence to the initially learned one.

### Sexually dimorphic circuits

Two genes coding for transcription factors, *fruitless* and *doublesex*, shape sexually dimorphic features of neural circuitry in flies, and regulate dimorphic patterns of social behaviour. Catalogues of *fruitless* and *doublesex*-expressing sexually dimorphic neurons [42–45] serve as the foundation to study the role of circuit components in sexual behaviour. Recent work compared a *doublesex^+^* neuronal cluster (termed aDN) between sexes [•46]. Using light microscopy (LM) images in males, they predicted that aDN neurons receive direct input from LC10 visual neurons that are required for orienting and tracking female movement during courtship. In females, connectomic analysis showed that aDN neurons instead receive olfactory inputs, and are required for selecting communal egg-laying sites. Whereas the male light microscopy (LM) analysis confirmed specific hypotheses, the female analysis enabled a comprehensive mapping of the network, discovering novel components, and generating unexpected behavioural hypotheses. In the near future, comparing male and female connectomes will link many known morphological changes to actual wiring differences between the sexes.

The female connectome has guided recent studies of circuits regulating female-specific behaviours: sexual receptivity, egg-laying and female aggression. Wang *et al*. [•47] and Wang *et al*. [•48] identified the descending neurons controlling male-acceptance and egglaying, and their upstream circuitry, linking mating status with egg-laying, and demonstrating multisensory integration such as egg-laying substrate and the male courtship song. *fruitless*^−^*doublesex*^+^ pC1 neurons regulate both sex-specific female behaviours: they promote virgin receptivity by directly activating vaginal-plate-opening descending neurons, and block virgin egg-laying by indirectly inhibiting oviposition-descending neurons. Mating status is incorporated into the circuit via abdominal ganglia neurons, indirectly inhibited by male sex-peptide transmitted during copulation. These neurons activate the pC1a subtype, suggesting pC1 neurons contribute to a switch between an internal state of virgin female receptivity and mated egg-laying.

The circuitry for female aggression was also studied using the connectome [•49,•50]. aIPg neurons bidirectionally control female aggression and their reconstruction in the brain [••3,^16^] found reciprocal connections with pC1d, a pC1 subtype that promotes persistent aggression. Strong pC1 subtype interconnectivity might shift a female’s decision between aggression and receptivity, promoting an internal state according to social context and needs.

Our group used the female connectome to dissect circuits processing the male pheromone [•51]. We identified a novel olfactory projection neuron population responsible for the previously described effects of male pheromone on female sexual behaviour. A single olfactory channel diverges into two ‘labelled lines’ in the second neuronal layer, each with unique responses to a male and sex-specific behavioural effects. The third-order circuitry then fans out to tens of cell types, some of them specifically responding to distinct aspects of the signal, such as a speed-sensitive sensor for male approach, and a multimodal sensor integrating male smell and taste. This architecture produces detectors for specific social contexts, each with a dedicated pathway to promote an appropriate behavioural response.

### Navigation and the central complex

The brain’s navigation system has been a highly influential area of neuroscience for decades. Recent studies in insects have finally revealed detailed circuit mechanisms [52–54], inaccessible in vertebrates, and connectomics has played a key role.

The fly’s sense of direction emerges from integrating sensory and motor signals in the ellipsoid body (EB), generating an interconnected network, operating as a ring attractor. This circuit relies on combining both internal self-motion cues (angular velocity) [55,56] and external cues (polarised light, visual landmarks, mechanosensory wind cues or others), carried by ring neurons. Ring neurons form all-to-all connections with sensory-channel specificity, and connect to compass neurons (termed EPG [56]). Plastic connectivity from ring to EPG neurons allows the compass to learn and maintain heading in response to a stable sensory scene, and to quickly remap new scenes [57,58]. Connectomic analysis confirmed the basic architecture and identified additional ring neuron subtypes that provide diverse sensory input, supporting the emerging view of EB as multimodal cue integrator [••3,^59^–••61]. How are sensory cues prioritised? Connectomic analysis provided multiple insights. First, relative synaptic weights differ between various ring neurons onto EPG neurons. Second, there is a consistent hierarchical organisation of inputs along EPG dendrites, such that self-motion inputs are closest to the root and are probably the most influential, followed by mechanosensory, visual and finally sleep-related ring neurons. Third, the authors found a hierarchy in the structure of mutual inhibition across ring neuron types, which could privilege salient sensory stimuli among others [••61]. Altogether, these results suggest that prioritising sensory cues for navigation could be hard-wired, reflecting cue reliability and the species’ evolutionary needs.

Flies are able to integrate their own turns along a path to compute the travelled journey and estimate a straight vector back to the starting point. This process, called path integration, requires a constantly updating vector computation of movement direction and speed, relative to a target location. While path integration is best known for central place foragers such as bees and ants [62], it was also confirmed in *Drosophila* [63], inviting connectomics circuit dissection. Path integration is suggested to be computed in another region of the central complex, downstream of the EB, the fan-shaped body (FB). Analysis of the hemibrain revealed a densely recurrent architecture of FB interneurons, tiling either horizontal layers or vertical columns, suggesting that columnar FB neurons are phase-shifted in their anatomical heading angle relative to neighbouring cells. These repeated connectivity motifs and phase shifts, combined with self-motion and head-direction inputs, could support vector-based navigational computation in the FB, ideal to perform coordinate transformations necessary for path integration [••61].

Emerging experimental work strengthens the anatomically based models [••61,^62^]: flies indeed perform path integration when foraging [63], remember target location after it disappeared and when faced with two distinct vanished targets, flies centre their search at a location between them [64]. These results suggest that flies accumulate experience while foraging to develop an internal sense of the target centre — a coordinate system centred on the world, rather than the self. Two papers recently provided evidence that the FB network indeed encodes a travel vector, by combining heading information from EB compass neurons with velocity pre-motor signals to perform vector computations [•20,•65]. Downstream, this body-centred velocity vector is transformed to a world-centred velocity vector, which can serve as the basis for remembering a target location during navigation.

Connectomic analysis described additional FB input from multiple regions, for example linking the FB to the MB [••61], supporting the idea of the FB as a centre for context-dependent navigational control, enabling flexible regulation of behaviour [66]. Additionally, recent work described FB input from olfactory neurons, and demonstrated that olfactory context modulates turning behaviour in response to wind [67].

## Other perspectives

The trend in generating larger and denser connectomes will enable comparative connectomics in *D. melanogaster* to directly link plasticity during an animal’s lifetime to behavioural diversity and circuit architecture. Similarly, comparing connectomes across sex and developmental stages will teach us about processes, leading to network maturation and specification [68,69].

Despite the extreme morphological variability of insect species and their rich behavioural repertoires, insect brains have evolved from a common ground plan dating back 500 million years [70]. Recent connectomes of the central complex of bumble bees [71] and butterfly lamina [72] highlighted the usefulness of comparisons to *D. melanogaster*, demonstrating both conserved core circuitry and species-specific modifications. Even in vertebrates, the recent description of a zebrafish ring-attractor network with similarities to the fly [73], suggests great value in comparisons beyond insects. We expect that inter-species comparisons will directly identify neuroanatomical changes and conservation correlated with species differentiation and behavioural evolution.

## Box 1 Resources and challenges

Datasets:

- L1 larval central nervous system (CNS) [9]: published sparse connectome available via Virtual Fly Brain (https://l1em.catmaid.virtualflybrain.org/).
- Whole female brain, full adult fly brain (FAFB): sparse (https://temca2data.org/) and dense reconstruction communities (FlyWire, https://flywire.ai/) [••5,^16^]. Published sparse connectome data available via Virtual Fly Brain (https://fafb.catmaid.virtualflybrain.org/).
- Partial female brain, hemibrain: connectome data, including segmentation, image data and queries available via neuPrint (https://neuprint.janelia.org/) [••3,^74^].
- Female VNC, FANC: sparse connectome available via Virtual Fly Brain (https://fanc.catmaid.virtualflybrain.org/). Dense proofreading community (https://www.lee.hms.harvard.edu/phelps-hildebrand-graham-et-al-2021)
- Male VNC, MANC: connectome data will be available via neuPrint [74].

Tools for analysis or visualisation:

- Neuroglancer (https://github.com/google/neuroglancer): WebGL-based viewer for volumetric data, often used for EM connectomics projects.
- NeuronBridge (https://neuronbridge.janelia.org/) [27,•30]: resource allowing similarity matching between MultiColour Flp-out LM images and EM morphologies.
- natverse (https://natverse.org/) [37]: collection of R packages for neuroanatomical analysis, including light microscopy (LM) and EM connectomics data.
- NAVis and friends (https://navis.readthedocs.io/en/latest/source/other_libraries.html#) [37]: collection of Python packages for neuroanatomical analysis, with some overlap in functionality to natverse.
- Virtual Fly Brain (http://www.virtualflybrain.org/): *Drosophila melanogaster* neuroscience resource integrating published data with a rich vocabulary for cell types.
- Insect Brain Database (https://insectbraindb.org/) [75]: neuroscience resource for species, neuroanatomy and functional information for a wide range of non-*Drosophila melanogaster* insects.
- Fruit Fly Brain Observatory (https://www.fruitflybrain.org/) [76]: ecosystem of tools to explore *Drosophila* neuroanatomical data, including morphology, connectivity and physiology, with the aim of generating networks and models.
- Brain Circuits (https://braincircuits.io/): website listing available EM connectomics and light microscopy (LM) libraries and atlases for vertebrates and invertebrates, together with available tools. Also provides own analysis tools for EM datasets.

Challenges:

1. Proofreading: a) ensuring a significant and consistent neuron, cell type or region completeness. This is affected by the proofreading strategy (distributed community or centralised), resources available, quality of image data and awareness of issues that impinge on morphology and synapse-segmentation outputs. Furthermore, the quality of the segmentation will depend on how representative the ground-truth data are with regard to the heterogeneity of image features almost always observed in bigger volumes. b) Storage and handling of larger EM volumes and segmentations, together with significant amounts of concurrent edits requires a robust but user-friendly proofreading and annotation system.
2. Neuron and cell type identification: obtaining whole-neuron morphologies is crucial for this step. Prior knowledge, leveraging other EM volumes and the ability to use automated morphological and connectivity-similarity methods [••3,•17,^36^] to compare new morphologies to previously identified single neurons or cell types, together with expert assessment, drives this process. The output quality and comprehensiveness of this process is significantly affected by the quality of the data being mapped and what it is being mapped to. On the one hand, there is the inherently sparse nature of light microscopy (LM) data for cell types and the incomplete descriptions in publications. On the other, the truncated and large number of new EM morphologies, the difficulty in assigning cell types and the lack of comprehensive inter- and intraindividual comparisons for both sexes. Common to both is the experience-dependent or -independent morphological and connectivity variability [•17,^31^,^77^]. This process of mapping cell types also leads to the identification of driver lines [••28,•29] that can be used for behavioural assays and the increase in the availability of sparsely labelled lines [78] is facilitating this process. However, similar but distinct cell types morphologically or connectivity-wise in EM are often not distinguishable in light microscopy (LM).
3. Mapping other types of data: features extracted from EM image data, such as neurotransmitter predictions, can be directly linked to a morphological entity, often classified at the cell type level (Figure 2). However, the small number of neurons known to express nonfast-acting neurotransmitters makes it difficult to collect enough ground truth data to generate confident predictions for a comprehensive set of neurotransmitters, currently identified by immunostaining methods. Linking EM to transcriptomics data is dependent on having expression markers for cell types as identified by light microscopy (LM) methods, thus only available for a small subset of types. The recent Fly Cell Atlas effort identified 80 clusters of neurons or glia types in the adult head, though the majority of neuron types in the central brain were unannotated [•21].
4. Stereotypy and its impact on the interpretation of the wiring diagram: the observed natural variability is also a factor on how we interpret circuit connectivity, and what behavioural changes it might cause, if any. Establishing robust ways of testing the links between observed connectivity and behavioural output, for a range of paradigms, contexts, internal states and so on, will help to clearly assess the impact of stereotypy.
